# Spatial pattern of invasion and the evolutionary responses of native plant species

**DOI:** 10.1111/eva.12398

**Published:** 2016-07-17

**Authors:** Gisela C. Stotz, Ernesto Gianoli, James F. Cahill

**Affiliations:** ^1^ Department of Biological SciencesUniversity of AlbertaEdmontonABCanada; ^2^ Departamento de BiologíaUniversidad de la SerenaLa SerenaChile; ^3^ Departmento de BotánicaUniversidad de ConcepciónConcepciónChile

**Keywords:** adaptation, conservation biology, evolutionary theory, invasive species, natural selection and contemporary evolution, species interactions

## Abstract

Invasive plant species can have a strong negative impact on the resident native species, likely imposing new selective pressures on them. Altered selective pressures may result in evolutionary changes in some native species, reducing competitive exclusion and allowing for coexistence with the invader. Native genotypes that are able to coexist with strong invaders may represent a valuable resource for management efforts. A better understanding of the conditions under which native species are more, or less, likely to adapt to an invader is necessary to incorporate these eco‐evolutionary dynamics into management strategies. We propose that the spatial structure of invasion, in particular the size and isolation of invaded patches, is one factor which can influence the evolutionary responses of native species through modifying gene flow and the strength of selection. We present a conceptual model in which large, dense, and well‐connected patches result in a greater likelihood of native species adaptation. We also identify characteristics of the interacting species that may influence the evolutionary response of native species to invasion and outline potential management implications. Identifying areas of rapid evolutionary change may offer one additional tool to managers in their effort to conserve biodiversity in the face of invasion.

## 1 Introduction

Invasive species can strongly impact native species diversity (Pyšek et al., 2012; Vilà et al., 2011) and ecosystem function (Pyšek et al., 2012; Strayer, 2012; Vilà et al., 2011; Weidenhamer & Callaway, 2010). Great efforts are made to control and eradicate invasive species (Roques & Auger‐Rozenberg, 2006; Simberloff, 2014), with both positive (Hoffmann, 2010; Wotherspoon & Wotherspoon, 2002) and negative outcomes (Rejmánek & Pitcairn, 2002). In spite of their strong negative impact on native species, and our limited ability to eradicate them, invasive plant species have not led to the global extinction of many native species, but this is thought to be a matter of time (Gilbert & Levine, 2013). The time lag between invasive species establishment and native species extinction risk gives native species a window of opportunity to evolve adaptive traits and thus persist within the newly structured community. There is growing evidence that some plant species can evolve in response to invasion (see Oduor, 2013 for a meta‐analysis on native species adaptation to invasion and Strauss, Lau, & Carroll, 2006 for a review). Strauss et al. (2006) reviewed 33 examples of native species evolution in response to invasion and argued that understanding when native species are more likely to evolve in response to invasion can help us to understand the long‐term impact of invasions. Native species evolutionary responses could facilitate the coexistence between native and invaders, therefore lessening the impact of invasive species on native plant populations (Strayer, Eviner, Jeschke, & Pace, 2006).

Taking advantage of evolutionary responses of native species to invaders may help manage the impact of invaders. Existing management strategies in response to invaders are diverse (Simberloff, 2014; Theoharides & Dukes, 2007) and can include the use of biocontrol agents, promoting intact native communities, and species removal. We may be able to use evolutionary responses of native species to refine and complement these currently used strategies. Strategies of early control have been relatively effective at reducing long‐term invasive species impact (Simberloff et al., 2013) and are generally more cost‐effective (Harris & Timmins, 2009) than control strategies in later stages of the invasion process. In contrast, efforts to control or eradicate long‐established invasive species have been less successful (Norton, 2009; Pala, 2008; Rejmánek & Pitcairn, 2002; Simberloff et al., 2013) and are typically more expensive (Panetta, 2009; Rejmánek & Pitcairn, 2002). It is particularly to those long‐established, highly abundant invasive species that native species may adapt (Thorpe, Aschehoug, Atwater, & Callaway, 2011). Evolutionary ecology has been an important tool in addressing other aspects of global change, such as delaying evolution of resistance in pests and pathogens and adaptation to climate change (reviewed in Carroll et al., 2014). Likewise, it could also be a useful tool when trying to manage or control invasive species (Leger & Espeland, 2010; Oduor, Yu, & Liu, 2015; Schlaepfer, Sherman, Blossey, & Runge, 2005).

Implementing the use of adapted genotypes of native species to complement current management strategies may to help minimize the impact of long‐established invasive species (Carroll et al., 2014; Schlaepfer et al., 2005; Strayer et al., 2006). Native species genotypes that have adaptations allowing increased coexistence with invaders could be used to increase the resistance of communities to further invasions (Schlaepfer et al., 2005), to minimize future extinction risks, to help manage the invader in already invaded communities, or to restore previously invaded areas. To implement this strategy, it is necessary to first understand under which circumstances, native species are more likely to show evolutionary responses to invaders.

Although many species are able to evolve in response to co‐occurring invasive species (Oduor, 2013), this is not always the outcome. Following the interaction with invasive species, some native species may evolve an increased ability to suppress the invader (Goergen, Leger, & Espeland, 2011; Rowe & Leger, 2010) or to better tolerate the presence of the invader through a reduction in competitive suppression (Callaway, Ridenour, Laboski, Weir, & Vivanco, 2005; Leger, 2008; Rowe & Leger, 2010), that is, evolution of character displacement to reduce competition when in sympatry (Brown & Wilson, 1956; Grant & Rosemary Grant, 2006). However, not all species are able to evolve in response to the interaction with a strong invader (Goergen et al., 2011; Mealor & Hild, 2007). For example, when testing for the adaptation of native species to the invasive cheatgrass, *Bromus tectorum*, it was found that native species were more tolerant to the invader in only two of four populations (Goergen et al., 2011). Identifying the conditions and processes influencing the likelihood of native species adaptation may help us to manage these eco‐evolutionary processes to improve our understanding of natural systems and complement current management strategies (Carroll, 2011; Schlaepfer et al., 2005).

Although the integration of evolution into the management of invasive species has been suggested earlier (Carroll, 2011; Leger & Espeland, 2010; Oduor et al., 2015; Schlaepfer et al., 2005), a more detailed eco‐evolutionary conceptual framework is needed to guide the development of both research and management practices for the control of invasive plant species. In this study, we first explore the requisites for, and evidence of, rapid evolution of native plant species in response to invasion. We subsequently propose a framework that focuses on using the spatial distribution of invasive species to understand the conditions under which native species are more likely to adapt to the pressures exerted by invasive species. We also discuss species characteristics and conditions that may influence the potential to respond to selective pressures. Finally, we outline potential management actions to promote rapid evolution, help control invasion, and prevent future extinctions due to invasion.

## 2 Factors Affecting Rapid Evolutionary Responses to Invaders

Evolution may seem slow over long periods of time; however, when selection is strong and constant, evolution can be rapid (Gómez‐González, Torres‐Díaz, Bustos‐Schindler, & Gianoli, 2011; Thompson, 1998). Plants are generally capable of evolving rapidly in response to local conditions (Bone & Farres, 2001; Leimu & Fischer, 2008). However, the evolutionary responses of plants to the interaction with neighbors remain poorly studied, compared to their evolutionary responses to other biotic and abiotic factors (Bone & Farres, 2001). As highlighted by Strauss et al. (2006) for plant–plant interactions to lead to an evolutionary response, there are at least three requisites: competitors must have an impact on neighbor fitness, fitness effects must be nonrandom (i.e., some genotypes more strongly affected than others), and the adaptive traits must be heritable (Futuyma, 2013; Strauss et al., 2006). Yet, plant–plant interactions occur over small spatial scales, where gene flow is highly likely and may prevent adaptation (Kawecki & Ebert, 2004). However, Turkington (1979) reported local adaptation of *Trifolium repens* to three different neighbors to have occurred not only over a short time period (10 years), but also over small spatial scales despite (highly likely) gene flow, which is possible when selection is strong enough (Richardson, Urban, Bolnick, & Skelly, 2014).

Many invasive species impose strong (and potentially novel) selective pressures on native species populations (Vilà et al., 2011). This may in part explain why most examples of rapid adaptation to neighbors come from interactions with invasive species (Lau, 2008; Oduor, 2013; Strauss et al., 2006), as strong selection is thought to be the main promoter of rapid evolutionary responses (Hairston, Ellner, Geber, Yoshida, & Fox, 2005). However, only native species with high levels of genetic variability in adaptive traits will be able to adapt in response to invasive species (Strauss et al., 2006). To coexist with invasive species, native plant species could evolve a higher impact on (competitive effect) or tolerance to (competitive response) the invader (Callaway et al., 2005; Goergen et al., 2011; Leger & Espeland, 2010; Rowe & Leger, 2010). Both, competitive effect and response, may be genetically determined and vary between individuals/genotypes (Baron, Richirt, Villoutreix, Amsellem, & Roux, 2015; Cahill, Kembel, & Gustafson, 2005; Johnson, Dinnage, Zhou, & Hunter, 2008; Willis, Brock, & Weinig, 2010).

Different traits may determine individual competitive ability, and this may depend on the context under which the interaction takes place as well as on the particular species/genotypes involved in the interaction (Baron et al., 2015; Wang, Stieglitz, Zhou, & Cahill, 2010). Some of the traits associated with an increased ability to suppress or tolerate invaders are as follows: earlier and faster growth, greater height, larger seed size, greater root growth or root‐to‐shoot ratio, and increased resistance to allelochemicals (Callaway et al., 2005; Goergen et al., 2011; Lankau, 2012; Leger, 2008; Mealor & Hild, 2007; Rowe & Leger, 2010; Turkington, 1979). The network of genes underlying these traits may slow or decrease the likelihood of an evolutionary response (Kawecki, 2008). The genetic correlation among traits may facilitate evolution if adaptive traits are positively correlated, but it can also constrain adaptation (Etterson & Shaw, 2001; Orr, 2000; Pigliucci, 2003). Despite the potential complexity behind competition‐related traits, many of these traits have shown rapid evolutionary responses (Bone & Farres, 2001).

In spite of the growing body of evidence of rapid evolution in response to plant–plant interactions, there is still some reluctance to integrate it into current conservation strategies (Kinnison, Hendry, & Stockwell, 2007). Although evolution is not always easy to detect, there are some indicators of which species/populations are more likely to evolve adaptations to persist in invaded areas. Linking evolutionary processes to observable ecological patterns and processes may (i) help to bridge the gap between evolutionary ecology and conservation biology and (ii) lead to the implementation of evolution‐informed management practices. Here, we propose a framework where spatial patterns of invasion can be used to predict the likelihood of native species adaptation to invaders.

## 3 The Spatial Pattern of Invasion

**Figure 1:**
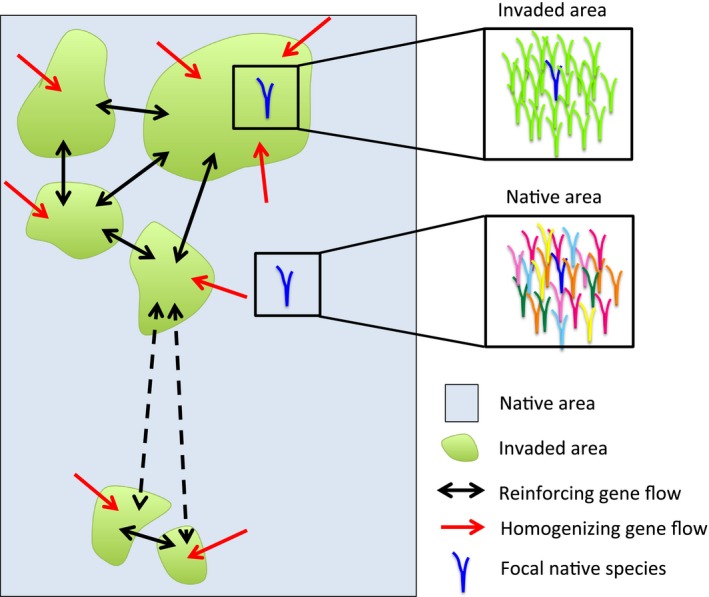
Conceptual diagram of the landscape pattern of invasion and the different kinds of gene flow affecting the adaptation of native species to invasion. Green areas symbolize invaded patches, the light‐blue area represents the matrix of native habitat, and blue individuals represent native species. As shown on the right part, a native individual in an invaded area interacts almost exclusively with the invader, while native individuals in native areas interact with several species. Red arrows stand for homogenizing gene flow (source: native plants from native areas), while black arrows stand for reinforcing gene flow (source: native plants from other invaded areas). Continuous‐line arrows indicate high rates of gene flow, while dashed‐line arrows indicate low rates of gene flow

By definition, invasive species are highly dominant (i.e., show high relative abundance and density) where they invade (Lowe, Browne, Boudjelas, & De Poorter, 2004; Richardson et al., 2000). Invasive species dominance is, however, not continuous across the landscape, as invaders may form patches or ‘islands’ of invasion (Fig. 1) (Kolb, Alpert, Enters, & Holzapfel, 2002; Lewis & Pacala, 2000). Their presence and dominance across the landscape may be limited, for example, by dispersal, disturbance, enemies, or abiotic conditions, leaving areas between invaded patches where native species persist (Fig. 1) (Huenneke, Hamburg, Koide, Mooney, & Vitousek, 1990; Kolb et al., 2002; MacDougall & Turkington, 2006). This spatial variation in dominance by invasive species may result in concomitant spatial patterns in the evolutionary responses to invasion. If true, we could use characteristics of the spatial pattern of invasion to predict where native species are more likely to be adapted.

Characteristics of the spatial pattern of invasion, such as patch size and distance between invaded patches, may determine the likelihood of native species adaptation to invasion. Just as size and distance from immigrant source were found to be major determinants of ecological and evolutionary processes in islands (Island Biogeography Theory; Losos & Schluter, 2000; Simberloff & Wilson, 1969; Simberloff, 1974), we believe that size and isolation of these ‘islands of selection’ are important determinants of eco‐evolutionary processes between native and invasive species (Leger & Espeland, 2010). Likewise, size and isolation among areas with different selective pressures are key factors in the evolution of insecticide resistance in pests and pathogens (Carrière et al., 2004; Gould, 2000; Sisterson, Carrière, Dennehy, & Tabashnik, 2005). For example, because of the widespread use of *Bt* crops (crops transformed to contain a transgene for an insecticidal protein), the evolution of resistance in pests and pathogens is a concern. One of the strategies used to prevent the evolution of resistance is to plant non‐*Bt* cultivars as refuges for the survival of susceptible pests (Gould, 1988, 2000; Roush, 1994), which has proven to be a successful approach (Tabashnik, Brévault, & Carrière, 2013). Thus, short distances facilitate high gene flow between areas, and size or abundance of refuges allows for large enough population size of susceptible pest genotypes (Caprio, Faver, & Hankins, 2004; Carrière et al., 2004; Sisterson et al., 2005). Similarly, we propose that size and isolation of invaded areas may determine the likelihood of evolution of ‘resistance’ in native species against invaders.

## 4 The Influence of Patch Size and Isolation on Native Species Adaptation to Invasion

**Figure 2:**
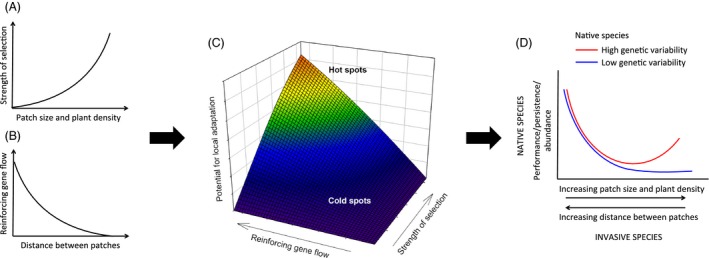
Conceptual model of eco‐evolutionary dynamics between native and invasive species as a function of size and isolation of invaded patches. The strength of selection is predicted to increase as a function of invasive species patch size and density (A). Reinforcing gene flow is predicted to decrease with distance between invaded patches (B). Consequently, the potential for native species adaptation would increase as patch size increases and the distance between patches decreases (C), resulting in cold and hot spots for the adaptation of native species to invasive species. If native species have the necessary genetic diversity to respond to selection, following an initial decrease of native species performance/abundance as the invasion process progresses (larger and closer invaded patches), there would be a recovery of those natives that succeed in adapting to the invasive species (D). However, if the genetic diversity of native species is too low, then an adaptive response to invasion and, therefore, the recovery of the population is unlikely (D)

The likelihood of an evolutionary response by native species to the invader will depend on the strength of selection, frequency of the interaction, and gene flow (Kawecki & Ebert, 2004; Strauss et al., 2006). Here, we argue that the spatial pattern of invasion, particularly the size and isolation of the invaded patches, may influence these processes, therefore altering the potential for native species adaptation (Fig. 2). Specifically, we propose that native adaptation is more likely to occur in large and well‐connected invaded patches, while in smaller and isolated ‘islands’, the selective pressure will be weaker and gene flow from noninvaded areas higher, thus decreasing the likelihood of adaptation by native species (Figs 1 and 2).

### 4.1 Invaded patch size and the strength of selection

Invaded patch size, or population size, is often associated with the invader impact on native species (Davies, 2011; Jackson, Ruiz‐Navarro, & Britton, 2014). Larger invaded patches will tend to have a higher density of invaders and reduced species diversity (Jackson et al., 2014). This would increase the likelihood of adaptation by increasing interaction frequency and consistency (less diffuse interactions) (Connell, 1980; Thorpe et al., 2011). Thus, in patches where a single invader becomes dominant, any individual would interact mainly, if not only, with the invader (Fig. 1). Larger patches and a higher density of invaders will also result in a stronger negative impact on native species performance (Jackson et al., 2014; Parker et al., 1999). Since the strength of selection increases with impact on fitness (Kingsolver et al., 2001), invasive species may exert stronger selection on native species in larger patches (Fig. 2a). Therefore, provided that native species have genetic variation for the selected traits and thus may show evolutionary responses to the selective pressures imposed by the invader (Strauss et al., 2006), then the likelihood of native species adaptation and persistence will be higher in larger and denser invaded patches (Fig. 2d) (Gomulkiewicz & Holt, 1995; Kinnison & Hairston, 2007).

### 4.2 Invaded patch isolation and gene flow

Native species adaptation will also depend on gene flow (Kawecki & Ebert, 2004). As invaded patches are often surrounded by a matrix of native species, gene flow among these areas is probable (Fig. 1). Gene flow can facilitate or hinder local adaptation, depending on its strength and origin (Kawecki & Ebert, 2004; Strauss et al., 2006). Gene flow tends to increase variation within populations, which is necessary for natural selection to occur. However, it may also reduce (or even prevent) selective processes in the population when individuals/genes arrive from areas with different selective pressures (Fig. 1) (Nosil, 2009; Riechert, 1993). In this case, if homogenizing gene flow from native areas is strong, it would limit or prevent adaptation of native individuals within the invaded patches. However, local adaptation can occur in the face of high gene flow, provided that the strength of selection is greater than the homogenizing effect of gene flow (Fitzpatrick, Gerberich, Kronenberger, Angeloni, & Funk, 2014; Kawecki & Ebert, 2004). For example, Fitzpatrick et al. (2014) found that adaptive phenotypic divergence of Trinidadian guppies in response to predators was maintained even after extensive gene flow. Similarly, local adaptation has been observed across small spatial scales, where gene flow is highly likely (reviewed in Richardson et al., 2014).

In contrast to homogenizing gene flow, reinforcing gene flow would facilitate local adaptation (Fig. 2) (Urban, 2011). Reinforcing gene flow is the arrival of individuals/genes from areas with similar selective pressures: in this case, from other invaded patches (Fig. 1). The arrival of pre‐adapted individuals/genes would facilitate adaptation of native species within invaded patches. Byars, Parsons, and Hoffmann (2009) found that genetic differences between high‐ and low‐ altitude populations of *Poa hiemata* were explained by biased gene flow: There was higher gene flow among populations at either altitude than across altitudes. Similarly, proximity among invaded patches would facilitate the arrival of preadapted individuals/genes (Thrall, Burdon, & Young, 2001; Urban, 2011) (Fig. 2B). This reinforcing gene flow could facilitate the adaptation of native species populations to the invader in those patches (Fig. 2C). Moreover, strong selection against mal‐adapted immigrants (Ehrlich & Raven, 1969; Lin, Quinn, Hilborn, & Hauser, 2008) can restrict the number and quality of immigrants, further limiting homogenizing gene flow.

### 4.3 Spatial pattern of invasion and a mosaic of adaptation

We propose that the likelihood of native adaptation to invasion is higher in large, dense, and well‐connected patches than in small, isolated patches (Fig. 2). In support of the importance of size and distance among interaction patches for evolutionary dynamics among species, studies on coevolution between pine trees and crossbills suggest that small, isolated forest areas tend to result in ‘cold spots’ for coevolution (see below), probably due to weaker selective pressures and higher homogenizing gene flow (Benkman & Parchman, 2009; Mezquida & Benkman, 2010), while the contrary would be true for large, dense, and well‐connected forest patches. However, although the potential for adaptation may be higher in large, well‐connected patches, the adaptive response of particular species will also depend on their genetic diversity, with a low genetic diversity potentially hindering an adaptive response (Fig. 2D) (Strauss et al., 2006).

The predictions of our model (Fig. 2) result in patches with native plants adapted to the invader and patches where such adaptation does not occur, as found by Goergen et al. (2011). This outcome is analogous to hot and cold spots for coevolution, as predicted by the Geographic Mosaic Theory of Coevolution (GMTC) (Thompson, 2005). GMTC integrates spatial mosaics of selection, the occurrence of coevolutionary hot and cold spots, and gene flow among these areas (Thompson, 2005). Viewing invaded areas as a mosaic of cold and hot spots for native species adaptation and/or coevolution could help us to better understand the dynamics of adaptation in these systems. Although our model aims at predicting hot spots for adaptation, coevolution between native and invasive species is also possible and it is potentially more likely to occur in large, dense, and well‐connected patches (Lankau, 2012; Leger & Espeland, 2010; Turkington, 1989). Greater connectivity among invaded patches may facilitate gene flow between invasive species populations/patches, potentially promoting their evolutionary potential (Leger & Espeland, 2010). Further, if native species adapt to invaders more often in large, well‐connected patches, it is in those patches where we could expect to see a reciprocal evolutionary response by the invader.

Invasive species often have a high evolutionary potential (Matesanz, Gianoli, & Valladares, 2010; Richards, Bossdorf, Muth, Gurevitch, & Pigliucci, 2006), thus making coevolution a possible outcome. However, coevolution among native and invasive plant species has rarely been studied (Leger & Espeland, 2010), and therefore, convincing evidence has only been reported once (Lankau, 2012). Lankau (2012) found that the invader garlic mustard (*Alliaria petiolata*) responded to high density of native competitors with an increased investment in sinigrin, a toxic allelochemical. In response, a native species, when co‐occurring with high‐sinigrin garlic mustard, was more tolerant to the allelochemical. However, a decline in garlic mustard's sinigrin production has also been documented (Lankau, Nuzzo, Spyreas, & Davis, 2009), potentially due to the evolution of resistance in native plants and microbes to the chemical, rendering it ineffective. As such, invasive species evolution, or the coevolution between native and invasive species, may also facilitate coexistence among interacting species and not necessarily lead to an escalating dynamic of increased ‘aggressiveness’ between them (Oduor et al., 2015).

GMTC, rather than merely predicting the occurrence of hot spots for (co)evolution, focuses on how coevolutionary hot spots—which may differ due to selection mosaics—interact with each other and with coevolutionary cold spots through the remixing of adaptive traits, thus determining the outcome of the interaction across broader scales (Gomulkiewicz et al., 2007; Thompson, 2005). This framework could be applied equally to the interaction between ‘adaptive’ hot and cold spots. We know that selection differs between invaded and uninvaded areas, with individuals in invaded areas being selected for traits such as increased growth rate, advanced phenology, particular root architecture, and tolerance to allelochemicals, among other traits (Callaway et al., 2005; Goergen et al., 2011; Lankau, 2012; Rowe & Leger, 2010). However, as predicted by GMTC, selection may also vary between invaded patches due to different environmental conditions, interactions with other species and/or invasion history (Gómez, 2003; Lankau, 2012; Oduor et al., 2015; Parchman & Benkman, 2008; Salgado‐Luarte & Gianoli, 2012). As invasive species are often distributed across broad geographic areas, selection mosaics are highly likely. For example, the invader cheatgrass (*Bromus tectorum*) increased nitrogen cycling when invading cool desert areas, but decreased it when invading arid grasslands (Ehrenfeld, 2003), likely imposing different selective pressures on native species in those areas. Evaluation of this scenario is important for invasive species management as it may imply that there is no single genotype of native species that is able to resist and/or tolerate the invader, but rather that adaptations are context dependent.

Because invasive species management occurs at the landscape level, it is important to underscore that variation in species interactions and selection at the local spatial scale can affect large scale population and community dynamics (Gomulkiewicz, Thompson, Holt, Nuismer, & Hochberg, 2000; Hartvigsen & Levin, 1997). As models show, both the abundance and distribution of (co)‐evolutionary hot spots across the landscape can determine the adaptation dynamics for the metapopulation as a whole (Gibert, Pires, Thompson, & Guimarães, 2013; Gomulkiewicz et al., 2000; Hanski, Mononen, & Ovaskainen, 2011; Nuismer, 2006). Similar models could be used to predict the dynamics of adaptation of native species to invasive species.

## 5 Biotic and Abiotic Factors That May Influence Native Species Adaptive Potential

Several characteristics of native and invasive species as well as environmental conditions may influence the likelihood of an adaptive response by native species to invasion. We briefly discuss below some characteristics that have been identified as important in determining the invasive species establishment and impact or species evolutionary dynamics in general (Catford, Jansson, & Nilsson, 2009; Holsinger, 2000; Lavergne & Molofsky, 2007; Reznick, Bryant, & Bashey, 2002), while suggesting possible links with the size and/or isolation of invaded patches. Other aspects of species, such as population size, generation time, and other life‐history traits, are also known to influence the rate of evolution and have been discussed elsewhere (Andreasen & Baldwin, 2001; Bousquet, Strauss, Doerksen, & Price, 1992; Hartl & Clark, 1997; Kostikova, Litsios, Salamin, & Pearman, 2013; Rosenheim & Tabashnik, 1991; Smith & Donoghue, 2008; Willi, Van Buskirk, & Hoffmann, 2006).

### 5.1 Mating system of native species

Self‐pollination can be advantageous under stressful conditions (Barrett, 1996; Horandl, 2006). For individuals adapted to invaded areas, vegetative reproduction and self‐pollination could assure reproduction in the absence (or low density) of sexual partners (Lloyd, 1992; Morgan & Wilson, 2005) and increase the probability of production of offspring well adapted to persist in invaded areas (Antonovics, 1968). In invaded patches, native plants that favor self‐pollination over out‐crossing would reduce homogenizing gene flow, thus further increasing the likelihood of adaptation (Antonovics, 1968). Therefore, selfers could be more tolerant to the isolation in invaded patches. However, self‐pollination may also lead to reduced fitness (inbreeding depression, Charlesworth & Charlesworth, 1987), smaller effective population size, and genetic diversity, thus reducing the likelihood of an evolutionary response to other stressors (e.g., disturbance, see below) and increasing population extinction risk (Gomulkiewicz & Holt, 1995; Holsinger, 2000; Kamran‐Disfani & Agrawal, 2014; Kinnison & Hairston, 2007).

### 5.2 Common versus rare native species

Common species have the advantage of larger initial population size, but a decrease in population size may have greater negative consequences on these species compared to rare species (Lankau & Strauss, 2011). Rare species, with their lower population sizes, are likely to show lower genetic variation and inbreeding depression, which may limit their evolutionary potential and make them more prone to demographic stochasticity (Avery & Hill, 1977; Reznick & Ghalambor, 2001; but see Wares, Hughes, & Grosberg, 2005; Willi et al., 2006). However, rare species may be adapted to avoid pollen limitation and decreased reproductive output in low‐density situations (Eckert et al., 2010; Kunin & Shmida, 1997; Lankau & Strauss, 2011; Reznick et al., 2002). Further, rare species may be better adapted to compete against inter‐ rather than intraspecific competitors, compared to common species (Shaw, Platenkamp, Shaw, & Podolsky, 1995). This may explain why, in certain cases, invasive species have lower impact on rare species (Bennett, Stotz, & Cahill, 2014; Powell, Chase, & Knight, 2013). When facing the strong selection expected in large, dense invaded patches, which often leads to significant reductions in population size, rare species—unless in very low densities—could be less affected than common species.

### 5.3 Invader's genetic diversity and multiple introductions

Genetic variation and repeated introduction of invasive species are known to influence their evolutionary potential (Lavergne & Molofsky, 2007; Matesanz, Horgan‐Kobelski, & Sultan, 2014; Vellend et al., 2007), but these factors may also affect the likelihood of native species adaptation. First, increased beta‐diversity of invader genotypes, over time and/or space, increases the variation of selective pressures on native species, potentially preventing an adaptive response (Aarssen & Turkington, 1985; Willis et al., 2010). Second, the presence of different invader genotypes across the landscape may decrease the rate of reinforcing gene flow (Fig. 1), as native species’ propagules from one invaded patch may be maladapted to establish/persist in another patch. The invader garlic mustard (*Alliaria petiolata*) varies in its levels of sinigrin (a toxic allelochemical) and therefore also in its selective pressure on native species: high‐sinigrin garlic mustard populations select for a greater resistance to the loss of arbuscular mycorrhizal fungi colonization in a co‐occurring native species (Lankau, 2012), while no such selection was observed in low‐sinigrin populations. These effects may be particularly detrimental for native species in isolated invaded patches, as the effective, functional distance among these islands will be far greater than the actual distance. In other words, multiple introductions of invasive plant genotypes could magnify the isolation effect. Although the arrival of new propagules of invasive species is generally regulated (Roques & Auger‐Rozenberg, 2006; Simberloff et al., 2013), this is not always the case for pasture and horticulture species, which are continuously bred and reseeded in their introduced range (Driscoll et al., 2014; Lonsdale, 1994; Reichard & White, 2001).

### 5.4 Disturbance

Invasive species are often facilitated by, or active drivers of, disturbance (Colautti, Grigorovich, & MacIsaac, 2006; Mack & D'Antonio, 1998; Sher & Hyatt, 1999). Disturbance may result in new sources of stress for native species and, in conjunction with invasion, may constrain a timely adaptive response by native species (Byers, 2002; Fakheran et al., 2010; Fenesi et al., 2015; Rolshausen et al., 2015). A recent meta‐analysis found that disturbance benefits invasive species, while native species are generally unaffected by disturbance in the presence of invasive species (Jauni, Gripenberg, & Ramula, 2015). In turn, native species that are affected by disturbance may be less likely to adapt to invasive species, because disturbance and competition may exert opposing selective pressures (Fakheran et al., 2010). Theory suggests that high and low frequency of disturbance select for a ruderals strategy and stronger competitive ability, respectively (Grime, 1974). If highly competitive genotypes are eliminated from highly disturbed landscapes (Fakheran et al., 2010), the adaptation of native species to disturbance may constrain their adaptation to invasion and vice versa. In sum, disturbance would hamper adaptation of native species to invaders, seemingly regardless of the size and isolation of invaded patches.

### 5.5 Plant–soil feedbacks

Many invasive plant species are known to modify soil conditions where they invade, which can affect native species performance and competitive ability (Bever, 1994, 2003; Ehrenfeld, 2010; Suding et al., 2013). Invader‐driven changes in soil conditions have the potential to influence both the strength and direction of selection on native species and their adaptive response (Chanway, Holl, & Turkington, 1988, 1989; Ehlers & Thompson, 2004). Further, these invader‐driven changes in soil conditions may cancel out the local or home advantages that native species may have had over invasive species (Byers, 2002) and further constrain their evolutionary responses (Gonzalez & Bell, 2012). This would be particularly true in large patches of invaders, where their greater abundance or density will bring about greater changes in soil conditions.

### 5.6 Enemy release

Many invasive species escape their natural enemies, experiencing reduced damage in the introduced range (Agrawal et al., 2005; Keane & Crawley, 2002). Native species able to persist in invaded patches may benefit from the association with invaders and also experience reduced damage (i.e., associational resistance, Barbosa et al., 2009). This reduced damage may lead to the re‐allocation of resources toward an increased competitive ability in native species, as with invasive species (Blossey & Notzold, 1995) favoring the adaptation of native species to coexist or compete with an invader. For example, the increased competitive ability of *Solidago altissima* after being experimentally released from aboveground herbivores occurred within 12 years in its native range (Uesugi & Kessler, 2013). The benefits of associational resistance for native species should be more evident in large patches of invaders, where natives would be more sheltered.

## 6 Management Implications

As management practices move forward, it is important to understand the eco‐evolutionary dynamics between native and invasive species. This information could improve current control strategies for invasive species. Testing whether native species are able to adapt to coexist, or resist, invasive species was a first step. Now that we know adaptation is possible, a second step is to identify the underlying mechanisms in order to determine under which conditions, adaptation is more likely to occur. In order to do so, we need to identify under which conditions, adaptation is more likely (which we propose doing based on characteristics of the invasive species spatial pattern) and which native species are more likely to adapt (based on characteristics of the native species). Knowledge of the conditions where adaptation is more probable and which species are more likely to adapt can allow managers to (i) increase the likelihood of native species adaptation and (ii) facilitate the gathering of adapted genotypes to increase resistance to invasion and restore invaded areas (Table 1).

**Table 1:** Predictions of the proposed framework, examples of methods that could be used to test the predictions, and management implications if predictions are correct

Predictions	Methods	Management implications, if predictions are correct
Large (dense), well‐connected patches are more likely to result in native species adaptation to invaders	Determine the strength of selection on key traits as a function of patch sizeUse molecular marker data to infer gene flow among subpopulations of native species in invaded areasQuantify adaptation across patches of different sizes and isolation to test for the individual and interactive effects of patch size and isolationStudy plant traits underlying resistance/tolerance to invasive plants targeting native species individuals from large, well‐connected patches	Propagules to reclaim invaded areas or increase the resistance of communities to invasion should be collected from large, well‐connected invaded patchesThe size and isolation of patches could be managed to increase the likelihood of native species adaptation by eliminating isolated patches and by targeting smaller patches first (eliminating new invasion foci), especially when there are not enough resources to eradicate the entire invasive species populationGene flow between invaded areas could be facilitated to increase the rate of reinforcing gene flowBreeding programs could select for traits that enhance resistance/tolerance to invasive species, based on studies done on individuals from large, well‐connected patches
Selection imposed by the invader on native species varies across the invaded range due to changes in biotic and abiotic conditions	Quantify and compare whether, and how, selection on key traits in invaded and uninvaded patches changes along abiotic or biotic gradientsTest whether native species adapted to interact with the invader on one end of the abiotic or biotic gradient show the same fitness advantage when on the other end of gradient	Propagules from adapted native species should be used to reclaim or increase resistance in areas with similar biotic and/or abiotic conditions to the areas where they were collected
Abundance and distribution of hot spots determine the adaptation dynamics for the metapopulation of native species, where gene flow between cold and hot spots may influence the likelihood of adaptation	Create a model to predict metapopulation dynamics of adaptation based on selection parameters estimated from the previously mentioned experiments and invasive species abundance and distribution	The abundance and distribution of hot and cold spots for adaptation could be managed to increase the likelihood of adaptation in a greater number of patches. Cold spot abundance could be decreased by eradicating the invader from those areas or by promoting reinforcing gene flow to increase the chances of it becoming a hot spot. Eliminating cold spots decreases potential homogenizing gene flow and removes new foci of invasion

Attempts could be made to facilitate native species adaptation (Leger & Espeland, 2010) (Table 1). Modifying the spatial distribution of the invader may not be a realistic goal, but gene flow between invaded areas could be manipulated. Reinforcing gene flow could be increased through additions of adapted genotypes (seeds or whole plants) into invaded patches, particularly for self‐incompatible species (Table 1). This procedure would be especially important in the more isolated patches. Many factors need to be considered when developing management strategies. If feasible, the complete removal of an invasive species is often desirable (but see Carroll, 2011; Schlaepfer, Sax, & Olden, 2011). However, for cases in which resources are not enough to eradicate all patches of an invasive species, we offer an additional tool to managers: we suggest starting by eliminating isolated patches, which would not only prevent establishment of new invasion foci but would also remove patches where adaptation of native species is unlikely (i.e., cold spots). Furthermore, identification of the traits underlying native species’ increased resistance and/or tolerance to invasive plant species can help to select traits to increase the resistance of native communities (Funk, Cleland, Suding, & Zavaleta, 2008); this should target individuals in large, dense, and well‐connected invaded patches (Table 1). Information on which type of species (e.g., common vs. rare species, selfing vs. outcrossing species, annual vs. perennial) are more likely to adapt may further advance our understanding of the conditions under which native species are likely to adapt.

Management practices could also reduce the evolutionary consequences of further introductions of new genotypes by regulating the planting of different/new genotypes of forage and horticulture species, as well as the movement of invasive species within the introduced range (Driscoll et al., 2014; Oduor et al., 2015; Reichard & White, 2001). Also, reducing the frequency of anthropogenic or novel disturbances in areas where adaptation of native species is likely (i.e., large, well‐connected patches) would reduce the extinction risk of native species and potentially facilitate their adaptation. The reduction of disturbances may also include discontinuing the eradication of invasive species in certain areas to promote the adaptation of native species, as argued by Carroll (2011): He proposed protecting invasive plant populations in one region of Australia where selection resulted in the adaptation of a native insect to more effectively consume the invader seeds (Carroll et al., 2005). Those adapted insect populations could then be used to promote gene flow to poorly adapted insect populations in other regions of Australia to help control a recent and serious invasion of a closely related plant species.

We predict that native species are more likely to adapt to coexist or compete against invasive species in large, dense, and well‐connected invaded patches (Fig. 2). If so, preference should be given to large and well‐connected invaded patches when collecting propagules from adapted genotypes for management purposes (Table 1). Within these sites, preference should be given to native species with larger population sizes, as small populations may be still in the process of adaptation (Gomulkiewicz & Holt, 1995). Besides implementing the use of adapted genotypes to complement management strategies, it is advisable to first evaluate the occurrence of a selection mosaic across the introduced range of invasive species, as predicted by the GMTC (Thompson, 2005). Testing for selection mosaics implies comparing selection by the invader on key traits in similar‐sized invaded patches along biotic and/or abiotic gradients across the invaded area. Invasive species may select for different traits or trait values depending on biotic or abiotic conditions. If there is evidence of a selection mosaic, the source of native species propagules should ideally match the biotic and abiotic condition of the area targeted for management (Table 1). It may be argued that selecting and using only a limited number of genotypes for management efforts can be disadvantageous because low genetic variation is associated with decreased fitness (Leimu, Mutikainen, Koricheva, & Fischer, 2006) and increased susceptibility to new stress factors (Frankham, 1996; Gonzalez & Bell, 2012; Willi et al., 2006). However, selected adapted genotypes may have a higher probability of survival in invaded areas, increasing population growth and the probability of population persistence (Reznick & Ghalambor, 2001).

Native species adaptation in the invaded patches will partly depend on the abundance of and connectedness between cold and hot spots (Hanski et al., 2011; Thompson, 2005). We could modify the connectedness of invaded patches to facilitate adaptation, while preventing further expansion of the invader. This could be achieved by removing cold spots (an easier task, as those are the smaller patches), or by increasing reinforcing gene flow to increase the likelihood of cold spots becoming hot spots (Table 1). The more hot spots in the landscape, the higher the probability of cold spots becoming hot spots by extensive reinforcing gene flow (Gibert et al., 2013; Hanski et al., 2011; Shirley & Sibly, 2001).

Overall, by better understanding the conditions that facilitate native species’ adaptation to invasion and by being able to predict where native species are more likely to have adapted, we can take advantage of these eco‐evolutionary processes to manage invaded ecosystems and complement current management strategies to control invasive plant species.
